# Supplementary material for: Discovery of a Closterovirus Infecting Jujube Plants Grown at Aksu Area in Xinjiang of China
Source: Viruses. 2023 Jan 17;15(2):267. doi: 10.3390/v15020267 (PMC9958854; doi:10.3390/v15020267)
Supplement: Supplementary file 1 [file viruses-15-00267-s001.zip › Supplementary Table .pdf]

Table S1. Primers used for the amplification of genome sequences of PAmpV-Ju variants AKS15-17 and AKS15-20 from jujube sample AKS15.

Variant	Fragment[a]	Primer[b]	Sequence (5′-3′)	Position (nt)	Size (bp)
	5′ end	5'-Outer	CTAATACGACTCACTATAGGGCAAGCA GTGGTATCAACGCAGAGT (Kit provided)		
		5'-Inner	CTAATACGACTCACTATAGGGC (Kit provided)		
AKS15-17	5′ end17	5RACE1	AGGAGTAGTCTGACGAAAAGAG	840-862	840
	F17-2	861F1	TGGACAGAATAGGAGTAAGAAT	786-807	2545
		3308R1	CATGTAAGTCAATTGATCTCCAG	3330-3308	
	F17-3	3234F1	CATCTTTTGCCCAGAGCTTAT	3234-3254	2373
		5925R1	GGGACTGGAGTGAAAGTGTCT	5945-5925	
		5587R1(2)	CACAGGATCAGTCAGGGCAGT	5607-5587	
	F17-4	5830F1	GTTGTAGGTGCCACTTCGAG	5830-5849	2077
		7886R1	GTGTCAGCCGTTCCGTCCAAT	7906-7886	
	F17-5	7401F1	TGGCCGTAACTTCTCCAATCAGA	7404- 7426	2329
		9730R1	CTTACGGGCACATTCCAGG	9748-9730	
	F17-6	9268F1	CCGACGGAAACTAAAGCC	9268-9285	1185
		10453R1	TCTTACTTGTCGACAGAACTGGC	10475-10453	
	F17-7	10292F1	TGCCCAATTATAACACTGCACTAG	10254-10277	1950
		12209R1	CATTCCCATATTCTCTTTACCTACT	12203-12179	
	F17-8	11878F1	CTGAATATCTGGGTCGTATAC	11878-11898	2263
		14119R1	GGTAACGCTCCCGGACAAGAAGT	14141-14119	
	3′ end17	3O-RACE-F1	CGAACGGTCACAGCATCAGATT	13593-13614	552
		3In-RACE-F1(2)	CACAGCATCAGATTGGTACAGTT	13601-13623	
AKS15-20	5′ end20	5RACE2	TGATCCGACGTGTGAAGCTAAG	867-889	867
	F20-2	621F2	GGAATCTTCACAATCAAGTTCT	621-642	1950
		2552R2	GGTCTGCTGGCTATAGTGT	2570-2552	
	F20-3	2164F2	GGTCTGCAATTTCCAACACT	2164-2183	2188
		4332R2	GTGACAAGAGACTCAAGCAA	4351-4332	
	F20-4	4191F2	GGATAGGTTTTGGATGAGACTCT	4191-4213	3668
		7863R2	GAAGTATCAGCAGTTCCATCCAGT	7886-7863	
		7836R2(2)	CTTTCATGTCACTTTTTACCATT	7858-7836	
	F20-5	7236F2	ACGGGGAACTTGCTATAAAT	7326-7345	2395
		9701R2	GAGCACATTCAGAACGTAGT	9720-9701	
	F20-6	9244F2	TCAGACGAAAGTTGAAACCT	9244-9263	1194
		10438R2	TTCAAGGCTTTATTCGCTGACT	10459-10438	
	F20-7	10178F2	TGTCCTACCGAAGTCTCACCCAAT	10178-10201	1960
		12116R2	TCAGACGGATCTTTGTTGCGAG	12137-12116	
	F20-8	11888F2	GATTGTGGGCGGGTGGATAT	11888-11907	2217
		14086R2	GACAAGAAGTGTGTGATACT	14105-14086	
	3′ end20	3O-RACE-F2	CTTGGGAGGTTACATTCTAGAT	13657-13678	454
		3In-RACE-F2 (2)	GTCAATGGGTGTACAAAACTT	13755-13775	

[a] The fragments of variants AKS15-17 and AKS15-20 were marked by numbers 17 and 20, respectively.
[b] Primers marked with (2) are used as semi-nested primers of the corresponding outer primer set.

Table S2. RT-PCR detection of persimmon ampelovirus jujube leaf samples collected from Xinjiang Uygur Autonomous Region of China using five sets of primers listed in Table1.

Variety	Sample ID[a]	Symptom[b]	RT-PCR					
			Pol-F/R	HSP70-F/R	CP-F1/R1	CP-F/R[c]	CP-F2/R2	CP-F/R[d]
Huizao	AKS1	CRS	-	-	-	-	-	-
Z. jujube var. spinosa	AKS2	CRS	-	-	-	-	-	-
Huizao	*AKS3*	CRS	-	-	-	-	-	+
Z. jujube var. spinosa	AKS4	CRS	-	+	-	+	-	-
Huizao	AKS5	CRS	-	-	-	+	-	-
Z. jujube var. spinosa	AKS6	CRS	-	-	-	-	-	-
Jixinzao	AKS7	B, MF, CS	-	-	-	+	-	-
Junzao	AKS8	B, CS	-	-	-	-	-	-
Huizao	AKS9	M, MF	-	+	-	-	-	-
Junzao	*AKS10*	N	-	-	-	+	-	+
	AKS11	N	-	+	-	+	-	-
	AKS12	CS, B	-	-	-	+	-	-
	AKS14	CS, B	-	-	-	-	-	+
	AKS15	CS, MF	+	+	+	+	+	+
	AKS16	CS, B	-	-	-	-	-	+
	AKS17	CS, B	-	-	-	+	-	+
	AKS18	M, B	-	-	-	+	-	+
	AKS19	CS, B	+	+	+	+	+	+
	AKS20	M, B	-	-	-	+	-	-
Dongzao	AKS13	CRS	-	-	-	-	-	-
Junzao	YGQ21	CS, MF, M	+	-	-	-	+	+
	YGQ22	CS, MF, M	+	-	-	-	+	+
	YGQ23	CS, MF, M	-	-	-	-	-	-
	YGQ24	CS, MF, M	-	-	-	-	-	-
	YGQ25	CS, MF, M	-	-		-	-	-
	YGQ26	CS, MF, M	-	-	-	-	-	+
Huizao	YGQ27	CS, MF, M	-	-	-	-	-	-
	YGQ28	CS, MF, M	-	-	-	-	-	-
	YGQ31	CS, MF, M	-	-	-	-	-	+
	YGQ32	CS, MF, M	-	-	-	-	-	+
	YGQ33	CS, MF, M	-	-	-	-	-	-
	YGQ34	CS, MF, M	-	-	-	-	-	-
	YGQ35	CS, MF, M	-	-	-	-	-	-
	YGQ36	CS, MF, M	-	-	-	+	-	-
	YGQ37	CS, MF, M	-	-	-	-	-	-
	YGQ38	CS, MF, M		-	-	-	-	-
	YGQ39	CS, MF, M	-	-	-	-	-	+
	YGQ40	CS, MF, M	-	-	-	-	-	-
	YGQ41	CS, MF, M	-	-	-	-	-	-
	YGQ42	CS, MF, M	-	-	-	-	-	-

	YGQ43	CS, MF, M	-	-	-	-	-	-
	YGQ44	CS, MF, M	-	-	-	-	-	-
	YGQ45	CS, MF, M	-	-	-	-	-	-
Junzao	XHL1	CS, MF, M	-	-	-	-	-	-
	XHL2	CS, MF, M	-	-	-	-	-	-
	XHL3	CS, MF, M	-	-	-	-	-	-
	XHL4	CS, MF, M	-	-	-	-	-	-
	XHL5	CS, MF, M	-	-	-	-	-	-
	XHL10	CS, MF, M	-	-	-	-	-	-
Huizao	XHL17	CS, MF, M	-	-	-	-	-	-
	XHL18	CS, MF, M	-	-	-	-	-	-
	XHL19	CS, MF, M	-	-	-	-	-	-
	XHL20	CS, MF, M	-	-	-	-	-	+
Z. jujube var. spinosa	XHL6	CS, MF, M	-	-	-	-	-	-
	XHL7	CS, MF, M	-	-	-	-	-	-
	XHL8	CS, MF, M	-	-	-	-	-	-
	XHL9	CS, MF, M	-	-	-	-	-	-
Z. jujube var. spinosa	AYKL1	CS, MF, M	-	-	-	-	-	-
	AYKL2	CS, MF, M	-	-	-	-	-	-
	AYKL3	CS, MF, M	-	-	-	-	-	-
	AYKL4	CS, MF, M	-	-	-	-	-	-
	AYKL5	CS, MF, M	-	-	-	-	-	-
Total	62		4	5	2	12	4	15

[a] Samples were collected from five different locations in Xinjiang Uygur Autonomous Region of China as indicated by the first letters (AKS, XHL, YGQ and AYKL) in the sample names.
[b] M, mosaic; B, blotch; W, waterlogging spots; MF, malformation; CS, chlorotic spot; CRS, chlorotic ringspot; N, symptom unknown.
[c,d] Nested RT-PCR using RT-PCR products derived using primer sets CP-F1/ R1 and CP-F2/R2 as templates, respectively.
+, positive; -, negative.
JYMaV were analyzed previously by our group []. Except for samples in italic negative for JYMaV, other samples are positive for JYMaV.
