# Supplementary material for: Discovery of a Closterovirus Infecting Jujube Plants Grown at Aksu Area in Xinjiang of China
Source: Viruses. 2023 Jan 17;15(2):267. doi: 10.3390/v15020267 (PMC9958854; doi:10.3390/v15020267)
Supplement: Supplementary file 1 [file viruses-15-00267-s001.zip › Supplementary Figure.pdf]

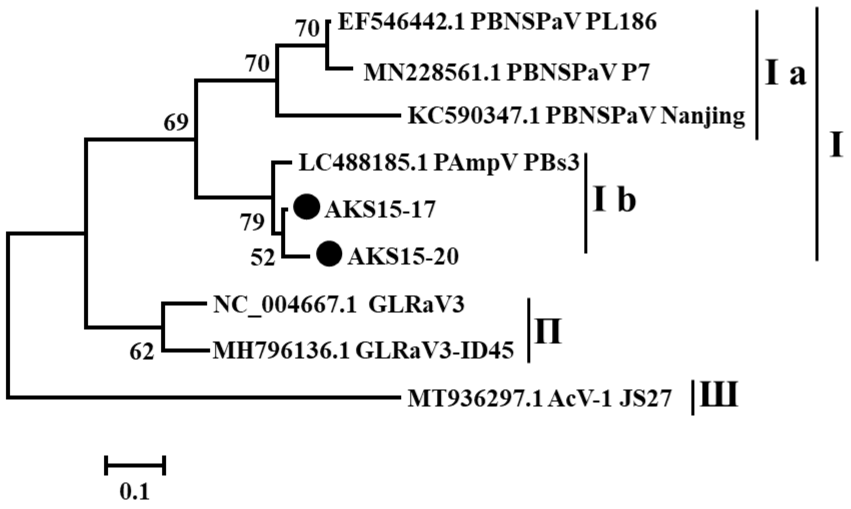

Figure S1. Phylogenetic tree constructed based on a conserved domain of oxygenase superfamily(2OG-FeII_Oxy_2) in polymerase of viruses in the family *Closteroviridae*. The phylogenetic tree was constructed using a neighbor-joining algorithm with 1000 bootstrap replications. Bootstrap support values >50% are shown at the nodes. PBNSPaV, plum bark necrosis stem pitting-associated virus; PAmpV, persimmon ampelovirus; GLRaV-3, grapevine leafroll-associated virus 3; AcV-1, actinidia virus 1.
